# Automatic screening for posttraumatic stress disorder in early adolescents following the Ya’an earthquake using text mining techniques

**DOI:** 10.3389/fpsyt.2024.1439720

**Published:** 2024-12-11

**Authors:** Yuzhuo Yuan, Zhiyuan Liu, Wei Miao, Xuetao Tian

**Affiliations:** ^1^ Collaborative Innovation Center of Assessment for Basic Education Quality, Beijing Normal University, Beijing, China; ^2^ Faculty of Psychology, Beijing Normal University, Beijing, China

**Keywords:** posttraumatic stress disorder, automatic screening, text mining, self-narratives, natural language processing

## Abstract

**Background:**

Self-narratives about traumatic experiences and symptoms are informative for early identification of potential patients; however, their use in clinical screening is limited. This study aimed to develop an automated screening method that analyzes self-narratives of early adolescent earthquake survivors to screen for PTSD in a timely and effective manner.

**Methods:**

An inquiry-based questionnaire consisting of a series of open-ended questions about trauma history and psychological symptoms, was designed to simulate the clinical structured interviews based on the DSM-5 diagnostic criteria, and was used to collect self-narratives from 430 survivors who experienced the Ya’an earthquake in Sichuan Province, China. Meanwhile, participants completed the PTSD Checklist for DSM-5 (PCL-5). Text classification models were constructed using three supervised learning algorithms (BERT, SVM, and KNN) to identify PTSD symptoms and their corresponding behavioral indicators in each sentence of the self-narratives.

**Results:**

The prediction accuracy for symptom-level classification reached 73.2%, and 67.2% for behavioral indicator classification, with the BERT performing the best.

**Conclusions:**

These findings demonstrate that self-narratives combined with text mining techniques provide a promising approach for automated, rapid, and accurate PTSD screening. Moreover, by conducting screenings in community and school settings, this approach equips clinicians and psychiatrists with evidence of PTSD symptoms and associated behavioral indicators, improving the effectiveness of early detection and treatment planning.

## 1 Introduction

Posttraumatic stress disorder (PTSD) is a tardive and persistent reactive psychiatric disorder that occurs after individuals have been exposed to abnormal threatening, traumatic, or catastrophic events (e.g., natural disasters, military combat, sexual assault, and witnessing the death of others) (1). There is substantial evidence that PTSD is associated with considerable impairment and difficulties that, if left untreated, may lead to subsequent depression, anxiety, substance abuse, conduct disorders, suicidal behavior, and decreased quality of life (see 2, for a review). Moreover, these negative effects may not be temporary; more than half of children and adolescents with PTSD symptoms will accompany them into adulthood (3), and approximately one-third of them are at risk of remaining with no hope of recovery throughout their lives (4). Specifically, at the cognitive level, PTSD patients typically show increased attentional bias toward traumatic events or threats (5). At the cerebral and neurological levels, patients present with some degree of morphological and functional changes in several brain regions, including the prefrontal cortex, hippocampus, and amygdala, such as reduced hippocampal volume and nonspecific lesions of prefrontal white matter (e.g., 6, 7).

In recent years, PTSD has attracted increasing research attention due to its various detrimental impacts on individual development (8–11). Studies have shown that effective psychological counseling and treatment can decrease the prevalence of PTSD, alleviate the severity of symptoms, and promote full recovery (12, 13). Rapid and accurate screening is precisely a prerequisite and key to achieving this goal. Therefore, it is necessary and urgent to conduct PTSD screening for at-risk individuals after experiencing traumatic events (14).

## 2 Literature review

### 2.1 Diagnostic criteria for PTSD

Since the American Psychiatric Association (APA) officially introduced PTSD to the Diagnostic and Statistical Manual of Mental Disorders, 3rd Edition (DSM-3) in 1980, it has gradually developed into one of the most widely accepted psychiatric disorders. According to the International Classification of Diseases (ICD-11) published by the World Health Organization, the Diagnostic and Statistical Manual of Mental Disorders (DSM-5), and the Chinese Classification and Diagnostic Criteria of Mental Disorders, 3rd Edition (CCMD-3), the most commonly reported symptom clusters associated with PTSD include “reexperiencing,” “hyperarousal,” and “avoidance”. Furthermore, the DSM-5 identifies “negative alterations” as the fourth most important symptom cluster of PTSD (15), which has become a widely accepted diagnostic criterion. As the diagnostic criteria for PTSD continue to be standardized, the measurement tools and methods are also evolving.

### 2.2 Traditional screening methods for PTSD

Currently, there are two main traditional methods for screening PTSD (16, 17). The first method employs self-report scales, preferred for their standardized format, ease of administration, and rapid scoring. However, these scales are limited to providing preliminary screening results (2). This limitation arises because people need to possess a certain level of introspection and reading comprehension to respond accurately to the items, which can be particularly challenging for younger populations, such as children and adolescents. Research has shown that self-assessment biases and varying levels of symptom awareness can significantly affect the accuracy of the data collected (18–20), suggesting that self-report scales may not always provide a complete picture of an individual’s PTSD status (21).

The second method involves structured interviews conducted in clinical settings, where clinicians assess PTSD by identifying traumatic experiences and symptoms described by individuals according to diagnostic criteria. The use of open-ended questions in the interviews allows patients to express their traumatic history, physical symptoms, and psychological state in their own words more freely and comprehensively. Existing evidence has revealed that the language patients use to describe their experiences serves as an important medium. Researchers and clinicians could gain insight into the symptoms and behavioral characteristics of patients from patients’ self-narratives, which are highly informative for the early detection of mental disorders (e.g., 22–25). Therefore, clinical interviews could provide a deeper understanding of PTSD symptoms and behavioral manifestations, thereby improving diagnostic accuracy. However, using clinical interviews to screen for PTSD also presents several challenges. First, the effectiveness of clinical interviews is highly dependent on the clinician’s own expertise and experience (26). Second, one-on-one interviews between participants and clinicians are time-consuming and costly, making them less feasible for large-scale screening. Third, individuals from diverse backgrounds (educational level, social status, living conditions, etc) may use different words to describe the same concept, leading to difficulties in mapping synonyms to standardized terminology and extracting consistent information that accurately represents the same domain (27). In addition, unlike the structured data obtained from self-report scales, the textual data obtained from interviews are often unstructured, complicating direct analysis using traditional statistical and measurement models (28).

### 2.3 Automated screening methods for PTSD using text mining techniques

Over the past decade, rapid advancements in natural language processing (NLP) and text mining have shown great potential in automatically detecting clinical information from unstructured free-text documents and converting it into structured data (27). Among these techniques, text classification (TC) is a supervised learning method that assigns a document to one or more predefined categories based on its content (29). TC has shown remarkable performance in a wide range of classification tasks across different domains (e.g., 30, 31). Given that individuals’ speech and writing pattern offer valuable clues about their emotional and cognitive states (32–34), numerous studies have applied text mining techniques to predict and identify risk indicators for mental disorders, such as depression, suicide, substance abuse, PTSD, and neurodevelopmental disorders (35–40), providing new tools and strategies for the screening, prevention, and intervention of mental health disorders.

Existing studies using text mining and machine learning for PTSD screening, data is primarily sourced from two main channels: social media data and publicly available datasets containing text transcripts of psychiatric interviews. By analyzing text data from various online forum users, Todorov et al. (41) found that individuals with PTSD used more singular first-person pronouns and fewer plural first-person pronouns, reflecting increased self-focus and reduced attention to others. Using two open questions (i.e., traumatic events and symptom description), He et al. (42) collected self-narratives from 300 participants and reported that individuals who have experienced multiple traumatic events tend to use more event-related terms (e.g., “fire,” “rape”) and temporal expressions (e.g., “year”), whereas individuals who have experienced a single traumatic event use more symptom-related terms (e.g., “flashbacks”, “nightmares”).In a subsequent study, He et al. (28) applied the n-gram representation models in conjunction with four machine learning algorithms in PTSD screening. The product score model with unigrams attained the highest prediction accuracy (82%) when compared with practitioners’ diagnoses. These studies highlight significant differences in the language patterns of self-narratives between individuals with PTSD and the general population based on their online data, which show some potential for identifying PTSD based on online text data.

Collecting PTSD-related text data from social media typically entails posing open-ended questions (e.g., What are the events that caused you most problems? What are their major impacts to your daily life? Would you please share your story)? or collecting trauma and daily life narratives shared by users in online forums. However, this approach is overly broad and fails to systematically guide individuals to adequately recall and report their traumatic experiences and PTSD-related symptoms, potentially leading to the exclusion of crucial diagnostic details. In addition, these types of narratives may result in a large amount of irrelevant information, complicating data cleaning for researchers and limiting the performance of PTSD prediction models. Furthermore, some studies that collect and analyze data directly from social media have not been fully addressed the ethical and legal implications of using this data in automated mental health screening. Issues such as data ownership, data anonymization, and the balance between beneficence and respect for patients’ autonomy are of particular concern (40).

Considering the limitations of freely expressed self-narratives on social media, transcripts from psychiatric interviews or responses to open-ended questions that simulate clinical interviews with informed consent, could offer a more reliable alternative for PTSD screening. Especially in psychiatric interviews, transcripts are often the primary source of information for psychologists, as they are easier to collect, require less preprocessing, and are incomparably easier to obtain informed consent from participants than audio or visual data (43). Moreover, most studies of automated screening for PTSD have developed text classifiers that only classify individuals into two distinct groups: PTSD and non-PTSD (44). This binary classification revolves around one single question, “Does this person have PTSD?”. This simplified approach offers limited information for clinicians, making it challenging to confirm a diagnosis and develop comprehensive treatment plans. Furthermore, this limitation often places clinicians in a difficult position, especially when the validity of their diagnosis of PTSD and related symptoms is challenged, fearing that the diagnosis could stigmatize patients or harm rapport with trauma survivors (26, 45). In this context, automated identification of specific symptoms and behavioral indicators of PTSD may be needed to augment diagnosis of PTSD by clinicians. Such information can also serve as objective evidence for PTSD screening, allowing clinicians to cross-check machine screening results, which enhances the reliability of the whole screening process.

### 2.4 The present study

**Figure 1:**
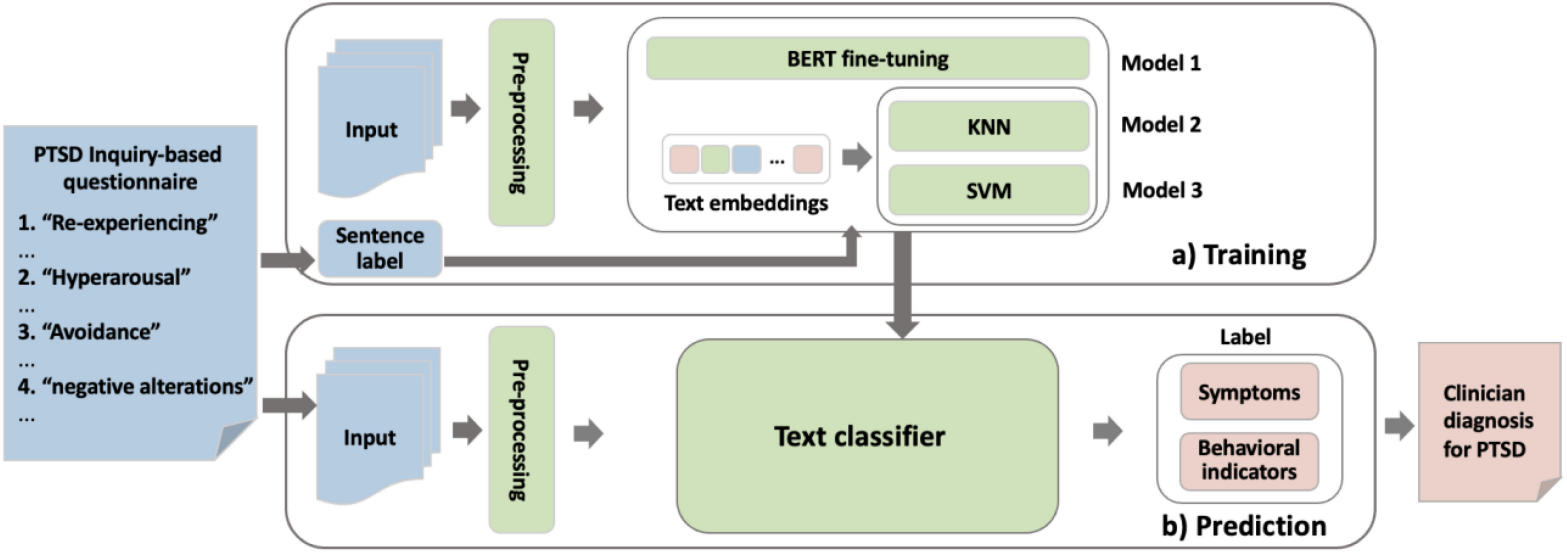
The overview of automated screening procedure for PTSD.

This study aimed to develop an automated screening method for screening patients with PTSD. Specifically, this study began by developing an inquiry-based questionnaire consisting of open-ended questions that simulated the questioning style of the clinicians during the face-to-face diagnostic interview, and participants were gradually guided to express their symptoms and experiences through a question-and-answer approach, thereby obtaining more standardized self-narratives and laying the foundation for subsequent accurate prediction of specific symptoms. Second, natural language processing (NLP) and text mining techniques were applied to construct an automated classification model for the textual responses of participants, thus enabling rapid screening of PTSD, in which a sentence-level text classification method was used to analyze texts with a finer granularity (46), providing a more effective basis for clinical diagnosis and later treatment. In this study, the automated screening method consists of three stages — data collection and preprocessing, model training and model prediction (see **Figure 1**).

## 3 Method

### 3.1 Development of the inquiry-based questionnaire

To ensure the reliability of the inquiry-based questionnaire, this study designed it according to the diagnostic criteria of the DSM-5 and imitated the methods of clinical psychologists’ structured interviews. The participants were asked to answer them one by one to collect their textual writing. Specifically, the inquiry-based questionnaire consisted of two parts and contained a total of five open-ended questions. First, participants were asked to briefly describe the traumatic event they experienced to evoke their memories of traumatic experiences and to ensure that they were more truthful and engaged when expressing their symptoms later. As this was not part of the examination of PTSD symptoms, the text obtained from this question was not included in the subsequent data analysis. Second, the remaining items were designed following the four clusters of PTSD symptoms defined by the DSM-5 and asked participants to describe whether they had any of the following problems and how they affected them: “It makes me have a lot of sad thoughts, memories or nightmares, and I can’t get rid of them”, “I hate to mention or be exposed to anything related to it”, “It gives me a lot of bad emotions and nothing can ever make me happy”, “It always makes me nervous and hard to calm down”.

### 3.2 Data collection and preprocessing

Prior to data collection, informed consent was obtained from both the participants and their guardians. The participants were informed that all data would be anonymized and used solely for academic research purposes. Furthermore, the participants were informed of the potential risks associated with the study, including the possibility of triggering traumatic memories related to the disaster, which could cause psychological distress. The participants could withdraw from the study at any time if they experience discomfort or distress.

430 survivors from grades 7-9 in a middle school in Ya’an, Sichuan Province, were invited to answer an inquiry-based questionnaire consisting of five open-ended items about their traumatic experiences after the earthquake. Meanwhile, participants were also asked to complete the PTSD Checklist for DSM-5 (PCL-5). As the PCL-5 has long been the most widely used screening tool for assessing PTSD symptoms by researchers and clinicians (47, 48), it was introduced in this study as a criterion to test the validity of the automated screening method. This scale consists of 20 items on a 5-point scale (0 = “not at all” to 4 = “extremely”). Confirmatory factor analysis using Mplus 7.11 revealed that item 7, “I would like to be exposed to something that reminds me of the earthquake” (after reverse scoring), had a negative factor loading on the “avoidance” dimension, while item 8, “My memory of the earthquake is vague”, had a very low factor loading on the “negative alterations” dimension. The poor psychometric indices of these two items may be because they are both reverse descriptions that are inconsistent with other items. After deletion, the PCL-5 displayed adequate internal consistency (Cronbach’s alpha = 0.93). The PCL-5 was developed based on the DSM-5, and its screening criteria were as follows: (1) the occurrence of “re-experiencing” or “avoidance” symptoms, with at least one item under the corresponding dimension scoring 2 or higher; (2) the occurrence of “negative alterations” or “hyperarousal” symptoms, with at least two items under the corresponding dimension scoring 2 or higher; and (3) the presence of all four clusters of symptoms meeting the criteria to determine PTSD.

The textual data were preprocessed. First, 69 participants who did not complete the PCL-5 were excluded. Second, 39 participants who either provided invalid responses or reported that they had not experienced the earthquake were excluded. Finally, 322 participants who had a clear description and complete expression of their experiences in the self-narrative text were retained, including 149 students in Grade 7, 128 students in Grade 8, and 45 students in Grade 9. After preliminary examination, it was found that the participants’ self-narratives did not correspond strictly to the clusters of symptoms that each open-ended question was designed to measure. For example, in response to the question aimed at measuring “avoidance” symptoms, participants also mentioned their performance regarding “negative alterations” symptoms. Therefore, participants’ self-narratives of the four symptom clusters were integrated to avoid possible classification errors arising from the above problems. Then, the self-narrative texts from 322 participants were preprocessed into sentences, and a total of 1,222 sentences were obtained.

### 3.3 Manual coding

Manual coding refers to tagging each sentence of participants’ responses to open-ended questions with PTSD symptoms and behavioral labels, which serves as the basis for model training and evaluation. The coding book was developed based on the PTSD diagnosis criteria of the DSM-5. The DSM-5 includes four primary symptom clusters of PTSD: “Reexperiencing,” “Avoidance,” “Negative Alterations,” and “Hyperarousal,” which were used as first-level codes. Under these four clusters, 20 behavioral indicators were used as second-level codes, as shown in **Table 1**.

**Table 1:** Comparison table of PTSD text coding and DSM-5 diagnostic criteria.

DSM-5 PTSD Diagnostic Criteria	First Level Code	DSM-5 PTSD Behavioral Indicators (Brief)	Second Level Code
A. Exposure to actual or threatened death, severe trauma or sexual violence in one of the following ways (or more):	01 Traumatic stimulation	A1. Directly experiencing a traumatic event	0101 Directly experiencing
		A2. Witnessing traumatic events that happen to other people	0102 Witnessing
		A3. Learn of a traumatic event that has happened to a close family member or close friend	0103 Learn of traumatic events that happened to family members or friends
		A4. Repeated or extreme exposure to the nauseating details of traumatic events	0104 Close exposure to traumatic events
B. After a traumatic event, there is one (or more) of the following invasive symptoms associated with the traumatic event:	02 Reexperiencing	B1. Repeated, involuntary and intrusive painful memories of traumatic events	0201 Repeatedly recalling traumatic events
		B2. Recurrent painful dreams with content and/or emotions related to traumatic events	0202 Recurrent dreams related to traumatic events
		B3. Dissociative reactions (e.g., flashbacks) in which an individual feel or acts as if the traumatic event is repeated	0203 A flashback or recurrence of traumatic feelings or actions
		B4. Intense or persistent psychological distress caused by exposure to internal or external cues that symbolize or resemble some aspect of a traumatic event	0204 Intense and persistent psychological distress when exposed to traumatic objects
		B5. A marked physiological response to internal or external cues that symbolize or resemble some aspect of a traumatic even	0205 Marked physiological responses to traumatic exposure
C. After a traumatic event, begin to continuously avoid the stimulation related to the traumatic event and have one or two of the following symptoms:	03 Avoidance	C1. Avoid or try to avoid painful memories, thoughts, or feelings about or highly associated with traumatic events	0301 Avoid painful memories or feelings associated with trauma
		C2. Avoid or try to avoid external cues that evoke painful memories, thoughts, or feelings about or highly associated with traumatic events	0302 Avoid external cues about traumatic events
D. Negative changes in cognition and mood related to traumatic events, which begin or worsen after the occurrence of the traumatic events, and contain the following 2 (or more) symptoms:	04 Negative Alterations	D1. Inability to remember an important aspect of a traumatic event	0401 Inability to remember an important aspect of a traumatic event
		D2. Negative beliefs and expectations about oneself, others or the world that continue to amplify	0402 Persistently exaggerated negative beliefs
		D3. Individuals blame themselves or others because of persistent cognitive distortions about the causes or consequences of traumatic events	0403 Blame oneself or someone else
		D4. Persistent negative emotional states	0404 Persistent negative emotional states
		D5. Significantly reduce interest in or participation in important activities	0405 Obviously little or no interest in participating in activities
		D6. A feeling of alienation or estrangement from others	0406 feel strange to others
		D7. Continuous inability to experience positive emotions	0407 Persistently difficult to experience positive emotions
E. Significant changes in alertness or reactivity related to traumatic events, which begin or worsen after the occurrence of the traumatic events, and contain the following 2 (or more) symptoms:	05 Hyperarousal	E1. Angry behavior and outbursts of anger	0501 Being irritable and attacking others or objects
		E2. Reckless or self-destructive behavior	0502 Reckless or self-harming behavior
		E3. Hyperarousal	0503 Hyperarousal
		E4. An excessive startle reaction	0504 An excessive startle reaction
		E5. Attention problems	0505 Difficult to concentrate
		E6. Sleep disorders	0506 Sleep disorders

Two graduate students specializing in clinical psychology, with a focus on trauma-related disorders, including PTSD, were recruited as coders for this study. Their prior clinical experience and involvement in mental disorder screening projects made them well-suited for this role. They were informed about the potential emotional impact of the task and were offered the option to withdraw at any time. They were also provided with access to the university’s psychological support services. Initially, the coders independently reviewed the narratives and then discussed their coding until they reached a consensus. Coding was conducted using Nvivo11.

Each sentence in the participants’ narratives was labeled based on whether it contained a PTSD symptom (first-level code) and its corresponding behavioral manifestation (second-level code). As mentioned above, the four symptom clusters presented in **Table 1** (first-level code 02-05 in columns 2, where 01 was an initial question used to elicit trauma-related experiences, and responses to this question were not used for symptom screening) and the 20 behavioral indicators (second-level code 0201-0506 in column 4) were used to code the self-narratives respectively. For example, a sentence was coded as “1” if it contained a behavioral indicator or symptom; otherwise, it was marked as “-1.” A sentence was marked as “0” if it contained only irrelevant information.

To ensure the reliability of manual coding, the proportion of agreement between the PCL-5 scale and the manual coding for symptom detection and PTSD diagnoses was calculated. A high agreement rate would indicate consistency between the narrative-based manual coding and the scale-based identification. The results showed that the agreement rate for the PTSD symptoms of “Reexperiencing,” “Avoidance,” “Negative Alterations,” and “Hyperarousal” were 67%, 77%, 76%, and 77%, respectively. The overall classification agreement rate between manual coding and the PCL-5 for PTSD diagnosis was 95%, indicating a satisfying level of agreement. The number of labels assigned to each sentence in the narratives of all participants is shown in **Table 2**.

**Table 2:** Coding results and sentence distribution.

First Level Code	Second Level Code	Total number of sentences
02		
	0201	29
	0202	31
	0203	10
	0204	108
	0205	15
Total		193
03		
	0301	52
	0302	30
Total		82
04		
	0401	4
	0402	31
	0403	3
	0404	150
	0405	10
	0406	4
	0407	35
Total		237
05		
	0501	55
	0502	3
	0503	68
	0504	19
	0505	7
	0506	17
Total		169
Irrelevant		543
	Total	1224

### 3.4 Text classification modeling

Sentence-level text classification models were constructed to automatically identify whether each sentence described the symptoms and behavioral indicators of PTSD. This study aimed to establish two classification models: a text classification model to predict symptoms (accurate to the first-level code) and a text classification model to predict behavior indicators (accurate to the second-level code).

Since the input data in this study consisted of unstructured text, the first step was to convert the text into feature vectors, a necessary process to allow for computational modeling. We employed two text classification approaches, resulting in the development of three classification models. In the first approach, we employed the Bidirectional Encoder Representations from Transformers (BERT) model to convert each sentence into a high-dimensional vector representation. These sentence vectors were subsequently used as input features for two widely-used machine learning classifiers: Support Vector Machine (SVM, 49) and K-Nearest Neighbors (KNN, 50). BERT, as a pre-trained transformer model, has demonstrated substantial efficacy in capturing contextual and semantic nuances within sentences, making it a powerful tool for text representation (51). Following vectorization with BERT, we trained the SVM and KNN classifiers to predict PTSD based on these text embeddings. SVM is a robust machine learning algorithm that has been widely used in text classification for its ability to handle high-dimensional spaces, while KNN, as a non-parametric method, is known for its simplicity and effectiveness with smaller datasets (49, 50).

The second approach involved fine-tuning the pre-trained BERT model directly on our dataset. BERT was initially trained on a large corpus of general text data from sources such as Wikipedia and BooksCorpus, providing it with a broad understanding of language structure. For our task, we adapted BERT by adding a classification layer and fine-tuning it specifically on our labeled PTSD data. This process allowed us to harness BERT’s deep contextual representation capabilities while optimizing it for the specific linguistic and contextual nuances of our dataset, enhancing its prediction performance for this domain-specific task (51). Fine-tuning enabled BERT to move beyond its general-purpose capabilities, addressing the particularities of PTSD-related language patterns within our data. BERT (bert-base-chinese) was trained using Python, while SVM and KNN were implemented using Matlab 2019a. The choice of these methods was driven by the relatively limited sample size in the dataset. Previous research has demonstrated that SVM and KNN are well-suited for smaller datasets, with both algorithms yielding reliable performance even when sample sizes are constrained (49, 50). In addition, fine-tuning BERT on our dataset allowed us to mitigate potential overfitting and generalization issues, which are common challenges in small data scenarios, by aligning the model more closely with our specific task requirements. Meanwhile, fine-tuning BERT allowed us to overcome potential generalization issues by tailoring the model to our specific task, further enhancing prediction accuracy.

To assess the generalization ability of the text classification model, a 10-fold cross-validation procedure was conducted as follows: the dataset was divided into 10 subsets, with the model being trained on nine and validated on the remaining one over 10 iterations. The average performance across all folds was used to provide a comprehensive evaluation of the model’s generalization. Four performance metrics—accuracy, precision, recall, and F1 score—were utilized to measure the model’s effectiveness. The calculation methods for these metrics are shown in **Table 3**.

**Table 3:** Contingency table for calculating classification metrics.

	having symptoms/behavioral indicators	not having symptoms/behavioral indicators
Classified as having symptoms/behavioral indicators	TP	FP
Classified as not having symptoms/behavioral indicators	FN	TN

TP, true positive; FP, false positive; FN, false negative; and TN, true negative.


$$Accuracy=\frac{TP+TN}{TP+TN+FP+FN}$$

$$Precision=\frac{TP}{TP+FP}$$

$$Recall=\frac{TP}{TP+FN}$$

$$F1=\frac{2\times Recall\times Precision}{Recall+Precision}$$

## 4 Results

### 4.1 Validation of classification models for first-level codes

The results revealed that the classification performance of the BERT model (accuracy = 0.732) was much better than that of the KNN model (accuracy = 0.494) and the SVM model (accuracy = 0.592); that is, 73.2% of the sentences in the test set were correctly classified by the BERT model.

In terms of various symptoms, as shown in **Table 4**, the classification results of the BERT model on four clusters of symptoms were good, and the F1 values ranged from 0.566 to 0.734. However, the classification results for all symptoms in the other two models were unsatisfactory, with F1 values ranging from 0.297 to 0.468 for the KNN model and from 0.400 to 0.601 for the SVM model.

**Table 4:** Text classification outcome indicators for symptom levels.

Model	First Level Code	Precision	Recall	F1 value
SVM	02 Reexperiencing	0.386	0.414	0.400
	03 Avoidance	0.660	0.552	0.601
	04 Negative Alterations	0.576	0.540	0.557
	05 Hyperarousal	0.618	0.580	0.598
KNN	02 Reexperiencing	0.276	0.322	0.297
	03 Avoidance	0.298	0.332	0.314
	04 Negative Alterations	0.416	0.534	0.468
	05 Hyperarousal	0.450	0.376	0.410
BERT	02 Reexperiencing	0.582	0.550	0.566
	03 Avoidance	0.724	0.742	0.733
	04 Negative Alterations	0.722	0.690	0.706
	05 Hyperarousal	0.704	0.766	0.734

### 4.2 Validation of classification models for second-level codes

The results showed that the classification performance of the BERT model (accuracy = 0.672) was better than that of the KNN model (accuracy = 0.426) and the SVM (accuracy = 0.552) overall; that is, 67.2% of the sentences in the test set were correctly classified by the BERT model.

In terms of various behavioral indicators, the results (see **Table 5**) showed that the BERT model produced better classification results than did the KNN and SVM models for almost all indicators, especially for indicators 0202, 0301, 0404, 0405, 0407, 0501, 0503, and 0506. The classification effects of the three models on behavioral indicators 0203, 0204, 0205, 0302, 0402, and 0504 were not satisfactory. To clarify the reason for this result, the original encoding file was examined, and it was found that some of the sentences that were coded as these behavioral indicators were identical. They may be labeled with different codes due to different contextual semantics, which makes it difficult for the models to perform appropriate classification. In addition, there were fewer than 10 sentences related to behavioral indicators 0401, 0403, 0406, 0502, and 0507, which represented 0 on all the performance metrics in the three models, so the corresponding results are not presented in the table.

**Table 5:** Text classification outcome indicators of the second level code.

Model	Second Level Code	Precision			Recall			F1 value		
		SVM	KNN	BERT	SVM	KNN	BERT	SVM	KNN	BERT
	0201	0.216	0.366	0.396	0.182	0.134	0.432	0.198	0.196	0.413
	0202	0.694	0.150	0.914	0.418	0.110	0.908	0.522	0.127	0.911
	0203	0.200	0	0.132	0.200	0	0.266	0.200	0	0.176
	0204	0.334	0.184	0.394	0.374	0.222	0.500	0.353	0.201	0.442
	0205	0.266	0.250	0.334	0.274	0.074	0.180	0.270	0.114	0.234
	0301	0.336	0.144	0.524	0.256	0.150	0.448	0.291	0.147	0.483
	0302	0.366	0.234	0.274	0.314	0.230	0.334	0.338	0.232	0.301
	0402	0.348	0.108	0.460	0.388	0.244	0.320	0.367	0.150	0.377
	0404	0.560	0.392	0.566	0.606	0.550	0.704	0.582	0.458	0.628
	0405	0.250	0	0.543	0.083	0	0.668	0.062	0	0.599
	0407	0.646	0.346	0.842	0.410	0.400	0.732	0.502	0.371	0.783
	0501	0.718	0.492	0.800	0.654	0.432	0.844	0.685	0.460	0.821
	0503	0.738	0.428	0.734	0.678	0.330	0.690	0.707	0.373	0.711
	0504	0.100	0.050	0.128	0.080	0.040	0.214	0.089	0.044	0.160
	0506	0.700	0.216	0.734	0.480	0.346	0.774	0.569	0.266	0.753

The meanings of second-level code are shown in **Table 1**.

## 5 Discussion

This study presents a novel automated screening method for PTSD based on self-narratives utilizing NLP and text-mining techniques. We developed an inquiry-based questionnaire that simulates a clinical structured interview based on the diagnostic criteria for PTSD in the DSM-5. Data were collected from 430 adolescent survivors of the Ya’an earthquake in China, who were asked to provide self-narrative responses about their experiences and emotions related to the four clusters of PTSD symptoms. Three classification models, KNN, SVM and BERT were used to identify the four symptom clusters of PTSD, as well as the specific behavioral indicators associated with each symptom, by analyzing individual sentences in the self-narratives. The results showed an overall accuracy of 73.2% at the symptom level and 67.2% at the behavioral indicator level, with the BERT classification achieving the highest accuracy. This method could enhance early detection by offering precise evidence of PTSD symptoms and related behavioral indicators in community and school environments. This information can support clinicians and psychiatrists in subsequent diagnosis and treatment planning with improved efficiency, accuracy and subtlety.

This study utilized text classification models to identify PTSD symptoms and behavioral indicators in participants’ self-narratives at the sentence level. To evaluate the performance of these models, we compared the consistency between manual coding and the text classification results. The results suggest that the BERT model outperformed KNN and SVM at the symptom level. Specifically, the accuracy for recognizing symptoms such as “avoidance,” “negative alterations,” and “hyperarousal” was relatively higher, while the performance for “reexperiencing” symptoms required improvement. Additionally, the classification accuracy at the behavioral indicator level was notably lower than that at the symptom level, and the classification results for several behavioral indicators did not meet expectations. The main reason for this discrepancy may be attributed to the small and uneven distribution of narratives containing symptoms and behavioral indicators in the current training samples. Moreover, identifying multiple symptoms and indicators within a single sentence constitutes a multi-label classification problem, where an increase in the number of label categories can generally lead to a higher probability of prediction errors (52). The large number of defined PTSD behavioral indicators, with the relatively sparse occurrence of sentences corresponding to these indicators in the dataset, resulted in less satisfactory classification accuracy.

Furthermore, the sample in this study consisted of students from schools in the earthquake-affected region, distinguishing it from previous studies on automated mental disorder classification that typically focused on clinical patients diagnosed with specific psychiatric disorder or control groups with other mental illnesses (e.g., 43) or PTSD patients who frequently seek help in online forums (28, 42). In contrast, this study aimed to identify potential PTSD symptoms in the general population, akin to mental health screenings in schools or community settings. Given the unique sample characteristics and limitations, the highest classification accuracies for symptoms and behavioral indicators were 73.2% and 67.2%, respectively. With an expanded training dataset, the accuracy in predicting both symptoms and behavioral indicators could be further improved.

In this study, two graduate students specializing in clinical psychology were recruited to code the participants’ self-narratives sentence by sentence. Both coders had received training in trauma-related disorders and had clinical experience. They developed a PTSD coding manual tailored to adolescent trauma self-narratives based on the DSM-5 diagnostic criteria for PTSD. Coders were tasked with reliably coding the sentences, which involved first identifying sentences that mentioned relevant symptoms, and then assigning symptom-related behavioral labels to each sentence based on participant descriptions. While individual clinician interviews would offer highly precise PTSD diagnoses, the large scale of 430 students presents significant challenges. Therefore, this study chose to assess the validity of the manual coding by examining its consistency with the commonly used PTSD screening tool, the PCL-5. Results showed a high level of agreement between the manual coding and the PCL-5 in identifying symptoms and diagnosing PTSD, indicating the reliability of the labeled dataset used for training the classification models.

Most previous studies utilizing text-mining techniques to detect mental disorders have relied on publicly available data, such as social, behavioral, and physiological health data obtained through social media, smart devices, and other sources (e.g., 32, 33, 53–55). Such data are characterized by their large scale and considerable noise, requiring extensive data cleaning before being input into models. Moreover, the ethical implications of using social media data remain a contentious issue. Some of these studies presumed implicit consent from users regarding the content they post to public platforms and directly analyze the data (e.g., 56, 57). However, this presumption overlooks that users may not expect and perceive their posts as public (58). In addition, despite previous studies on automated PTSD screening (28, 41, 42) gathering narratives posted in online forums with user consent, these narratives were few in number and rather generic. Unlike structured clinical interviews, the responses in these narratives were overly unbounded, resulting in self-disclosures lacking specific information about PTSD symptoms and behaviors.

Accurately predicting mental disorders depends on the ability to explore and extract the most discriminative features or patterns from large amounts of data. Therefore, in contrast to these studies based on public data and general narratives, one of the critical contributions of this research is to collect self-narratives explicitly linked to descriptions of PTSD symptoms. This approach simulates the structured interview process of clinical practitioners based on the DSM-5 diagnostic criteria for PTSD, aiming to enhance the accuracy and reliability of screening results by identifying symptoms and behavioral indicators of PTSD within text data. Furthermore, compared with the traditional self-report scale, the inquiry-based questionnaire proposed in this study guides individuals to describe their own experiences truthfully in a step-by-step manner without making judgments and choices about the items by themselves. Using self-narratives helps prevent the influence of individual response bias on measurement results, providing more specific and comprehensive information. As a result, the method developed in this study aids in the refinement of symptom identification, thereby better assisting clinicians in the early detection of potential patients and in designing targeted intervention and treatment plans for those exhibiting specific symptoms.

Existing studies on the prediction of PTSD based on text data have achieved classification accuracies higher than 0.8, suggesting the effectiveness and potential of automated screening tools (26, 28). However, these studies primarily focused on binary classification, distinguishing only between PTSD and non-PTSD, without providing predictions regarding specific symptoms or behavioral indicators. Building on previous research, this study extends the scope by attempting to identify internal PTSD symptoms and associated risk behaviors, provides a complete picture of the symptomatology exhibited by individuals with PTSD rather than dichotomy.

This study identifies PTSD symptoms and behaviors reflected in individuals’ self-narratives through linguistic content to some extent mirroring the structured interview process used by clinical practitioners. Nonetheless, it is also important to note that clinicians base their diagnose not only on “what” the patient says but also on “how” they express themselves. While the linguistic content, encompassing features like words, phrases and sentiments, has been shown to be a valuable asset for detecting mental disorders (59), acoustic features represent another promising data source (60). In fact, analyzing speech signals alone has shown the sufficient capability to diagnose PTSD automatically (61). Combining spoken audio with transcribed text significantly enhances the accuracy of predictive assessments compared to using either modality alone (62).

Multimodal assessments that incorporate speech, text, and non-verbal cues can rival the predictive accuracy of experienced psychiatrists (63). For instance, video recordings provide additional valuable information for mental health diagnosis, such as audio features and head postures related to speech patterns and expressions (64). Multimodal feature extraction and decision-level fusion methods also pave the way for robotic systems that can mimic clinicians in reviewing and recording individual vocal responses by integrating audio, visual, and textual data, leading to more comprehensive mental state analyses (64), facilitating the creation of more accurate predictive models.

However, audio and visual data are inherently more complex and demanding to process (43). In contrast, the textual data collected in this study, presented in the form of questionnaire, is much easier to obtain the participant’s consent, holding a major advantage in general clinical settings. Additionally, using written text rather than spoken language, which often involves incomplete sentences and requires the transcriptionist to determine sentence boundaries, can reduce errors and subjectivity (65). Furthermore, for PTSD patients, who may be vulnerable to anxiety or retraumatization from verbal expression, writing could be a more protective mechanism for recalling experiences (65). In summary, our text-based approach not only simplifies the collection of self-narratives in challenging clinical interview scenarios but also offers an efficient solution for PTSD screening in remote and underdeveloped regions that lack infrastructure and health care personnel.

In the aftermath of a major disaster, long-lasting psychological effects can vary and one of the most common psychological disorders is PTSD affecting around 33% of the population (66). In addition, approximately 25% of individuals may experience depression, including mental health workers involved in relief efforts (67). School-aged children and adolescents are particularly vulnerable, often exhibiting behavioral changes, fear, anxiety, recurrent intrusive memories of the disaster, and related problems such as learning difficulties, sleep disturbances, and somatic symptoms. Timely identification and intervention for at-risk individuals, especially mentally and physically immature adolescents, are crucial for preventing negative mental health and personality development consequences (68).

Building on previous studies, this research aims to develop a more fine-grained and automated screening method for PTSD to reduce burden on clinicians and resources. The proposed method is tailored for initial screening of PTSD in adolescent earthquake survivors, enabling early diagnosis and timely treatment planning through school counseling services and clinical hospitals. By extracting detailed symptoms and behavioral cues from self-narratives, clinicians can better interpret the data in a short period of time, enhancing the thoroughness and accuracy of early screenings, especially in disaster scenarios with limited resources and damaged infrastructure.

Some limitations of the present study should be mentioned. First, the PCL-5 was chosen as the criterion to test the validity of the inquiry-based questionnaire in this study, but self-rating scales cannot replace the clinicians’ diagnoses. A more ideal criterion would be clinical diagnosis by clinicians through face-to-face structured interviews, and future studies can improve on this basis and continue to advance the research. Second, although two graduate students in clinical psychology were responsible for the manual coding of self-narratives, they did so through discussion rather than independent coding. This is because this study has less textual data available for encoding. After the two coders agreed to form a stable coding framework, insufficient text remained to support independent coding. For the same reason, it is difficult to support using deep learning methods when the sample size is small. Future research can collect more samples and try to build more complex models. Finally, this study only screened for symptoms of PTSD in individuals who experienced the Ya’an earthquake in Sichuan Province. However, empirical studies have proven that not all trauma events will lead to the same performance (69), and the incidence of PTSD is related to the type and impact of disasters (70). Therefore, future research can construct models according to different trauma events, which may improve the prediction accuracy.

Furthermore, future research could integrate large language models (LLMs) to enhance initial screening for mental disorders. LLMs have seen increasing use in a variety of applications, offering the advantage of considering the broader context of text based on much larger parameters and datasets than conventional models, which typically analyze text by breaking it into individual sentences. Additionally, intelligent chatbots embedded with LLMs could be developed for PTSD clinical interviews. With proper informed consent and anonymization, patients could engage in free-flowing, interactive conversations with these chatbots, ensuring the ecological validity of AI in capturing potential risk factors. At the same time, multimodal data—including language, speech, gestures, and emotions—could be recorded for a more comprehensive analysis and diagnosis of mental health. This approach would not only reveal the underlying mechanisms of mental disorders but also improve the accuracy of screening and diagnosis.

## Data Availability

The raw data supporting the conclusions of this article will be made available by the authors, without undue reservation.
